# Parents' beliefs about appropriate infant size, growth and feeding behaviour: implications for the prevention of childhood obesity

**DOI:** 10.1186/1471-2458-10-711

**Published:** 2010-11-18

**Authors:** Sarah A Redsell, Philippa Atkinson, Dilip Nathan, A Niroshan Siriwardena, Judy A Swift, Cris Glazebrook

**Affiliations:** 1 School of Nursing, Midwifery and Physiotherapy, University of Nottingham, Queen's Medical Centre, B Floor, South Block, Nottingham, NG7 2HA, UK; 2 Nottingham City Primary Care Trust, 1 Standard Court, Nottingham. NG1 6GN, UK; 3 Nottingham University Hospitals NHS Trust, Nottingham, NG7 2UH, UK; 4 School of Health and Social Care, University of Lincoln, Lincoln, LN6 7TS, UK; 5 School of Biosciences, University of Nottingham, Nottingham, LE12 5RD, UK; 6 School of Community Health Sciences, University of Nottingham, Nottingham, NG7 2HA, UK

## Abstract

**Background:**

A number of risk factors are associated with the development of childhood obesity which can be identified during infancy. These include infant feeding practices, parental response to infant temperament and parental perception of infant growth and appetite. Parental beliefs and understanding are crucial determinants of infant feeding behaviour; therefore any intervention would need to take account of their views. This study aimed to explore UK parents' beliefs concerning their infant's size, growth and feeding behaviour and parental receptiveness to early intervention aimed at reducing the risk of childhood obesity.

**Method:**

Six focus groups were undertaken in a range of different demographic localities, with parents of infants less than one year of age. The focus groups were audio-recorded, transcribed verbatim and thematic analysis applied using an interpretative, inductive approach.

**Results:**

38 parents (n = 36 female, n = 2 male), age range 19-45 years (mean 30.1 years, SD 6.28) participated in the focus groups. 12/38 were overweight (BMI 25-29.99) and 8/38 obese (BMI >30). Five main themes were identified. These were a) parental concern about breast milk, infant contentment and growth; b) the belief that the main cause of infant distress is hunger is widespread and drives inappropriate feeding; c) rationalisation for infants' larger size; d) parental uncertainty about identifying and managing infants at risk of obesity and e) intentions and behaviour in relation to a healthy lifestyle.

**Conclusions:**

There are a number of barriers to early intervention with parents of infants at risk of developing obesity. Parents are receptive to prevention prior to weaning and need better support with best practice in infant feeding. In particular, this should focus on helping them understand the physiology of breast feeding, how to differentiate between infant distress caused by hunger and other causes and the timing of weaning. Some parents also need guidance about how to recognize and prepare healthy foods and facilitate physical activity for their infants.

## Background

The prevalence of childhood obesity in the UK has increased significantly since 1995[1], though more recent data suggests that rates have peaked and the incidence is leveling off[2]. The most recent combined rates for childhood overweight and obesity amongst 2-15 year olds are 31% for boys and 30% for girls[1]. Causes and consequences of childhood overweight and obesity have been investigated in a number of observational studies and three systematic reviews have concluded that rapid weight gain during infancy is associated with greater obesity risk in later life[3-5]. An infant's weight trajectory may be influenced by a number of risk factors that are potentially modifiable during their early years. These include infant feeding practices, parental response to infant temperament and parental perception of infant growth and appetite.

### Infant feeding practices

The relationship between breast feeding and obesity risk in childhood has been investigated in a number of studies, with contradictory findings[6]. A systematic review and meta analysis conducted in 2004, concluded that breast feeding has a small but consistent protective effect against the development of obesity during childhood[6]. There is emerging experimental evidence that the higher protein content of formula milk may be partially responsible for greater weight gain[7]. There is also observational evidence that early weaning onto solid foods (< 4 months) is significantly associated with overweight or obesity at 3 years[8]. A UK longitudinal cohort study found that infants fed with formula milk were introduced to solid foods earlier than breast fed infants and were less likely to have consumed fruit and vegetables[9]. These study results require cautious interpretation as the cohort consisted exclusively of white, singleton children, who were from more affluent backgrounds. There may be other differences between mothers who choose to breastfeed and those that do not (e.g. breastfeeding mothers may be more health conscious, more responsive to their infant and have a higher socio-economic status (SES)).

### Parental response to infant temperament

Parental response to infant temperament is a risk factor for childhood obesity[10]. A qualitative study of 14 low income mothers with overweight young children reported that food was used to soothe fussy infants, calm temper tantrums and shape behaviour[11]. In another study infants with a temperament dimension described as "fear" in relation to their acceptance or rejection of new objects or people were significantly slower to gain weight than those described as "distress to limitations" which is characterized by frustration, reduced sleep, excessive crying and fussiness. The authors speculate that parents who perceive their infants to be fussy or frustrated may use food as comfort which may account for the differences in weight gain between the two groups[12].

### Parental perception of infant growth and appetite

Parental perceptions about appropriate size, growth and feeding patterns play an important role in the development of childhood obesity. Baughcum and colleagues[11], in the study described above, reported that mothers had strong beliefs that heavy infants were desirable as weight was the best marker of health and successful parenting. In a later study, Baughcum and colleagues[13] assessed maternal feeding practices and beliefs using feeding questionnaires for infants (n = 453) and pre-school children (n = 634). They described different feeding behaviours between high and low income mothers. Low income mothers reported greater concern about their child's hunger, a lower tendency to use food to calm and greater difficulty feeding their children. They also reported pushing their child to eat more and engaging in more age-inappropriate feeding.

Parental beliefs and understanding are crucial determinants of infant feeding behaviour, therefore any intervention will need to take account of these views. This study aimed to explore UK parents' beliefs concerning their infant's size, growth and feeding behaviours and their receptiveness to early intervention aimed at reducing the risk of childhood obesity.

The objectives were:

• To explore perceptions of appropriate infant size and growth including possible cultural influences.

• To investigate parental views around infants identified as at risk of childhood obesity by a health professional.

• To determine parents' beliefs about infant feeding practices and weaning approaches

## Method

### Sampling and recruitment

Six study sites were selected with the intention of ensuring a higher probability of recruiting parents whose infants were at risk of developing obesity. The UK National Child Measurement Programme, which started in 2005, routinely measures children aged 4-5 years (Reception) and 10-11 years (Year 6),[14] and the intention was to use this data to select five study sites where the risk of childhood overweight and obesity was high and one site where the risk was low. The inclusion of a low risk site was to determine whether the views of these parents were vastly different from those parents who resided in a high risk site. Two urban sites with high rates of overweight and obesity were selected using this data (Table 1)[15]. Unfortunately National Child Measurement data was unavailable for the surrounding rural population. As social disadvantage is a risk factor for childhood obesity[8], the Index of Multiple Deprivation (IMD) (2007)[16] of the health centre location was used as a proxy measure for childhood obesity risk. Three further study sites were selected with high IMD scores and one with a low score (Table 1). Permission for the multi-centre study was obtained from the Nottingham Research Ethics Committee 1 and Nottinghamshire and Lincolnshire Primary Care Trusts (PCTs). The study was registered with the Primary Care Research Network (PCRN).

**Table 1:** Locality areas and IMD

Name of area		Description	Median (Range) IMD
Area 1	(n = 5)	City suburbs, average numbers of ethnic groups/low rates of childhood obesity (1)	12.19 (8.97-24.58)

Area 2	(n = 4)	Inner City, deprived, high numbers of ethnic group/high rates of childhood obesity (2)	32.92 (12.59-37.67)

Area 3	(n = 7)	Inner City, deprived, average numbers of ethnic groups/high rates of childhood obesity (2)	60.31 (35.57-66.29)

Area 4	(n = 9)	Coastal town, deprived, low minority ethnic groups/high rates of childhood obesity (1)	32.69 (10.39-41.39)

Area 5	(n = 8)	City suburbs, average numbers of ethnic groups/high rates of childhood obesity (2)	12.65 (10.97-28.23)

Area 6	(n = 5)	Rural town, low minority ethnic groups/high rates of childhood obesity (1)	23.01 (19.85-23.01)

(1) Area prospectively selected using IMD (2007) [16]

(2) Area prospectively selected using PCT data [15]

At each site, 8-10 parents attending child health (baby) clinics were approached by Health Visitors (UK specialist practitioners in public health) and asked to participate in the study. Inclusion criteria were that parents should be living in the study site and have an infant less than one year of age, during 2008-2009. One focus group took place in the local health or children's centre at each of the six study sites. Participating parents were offered a £20 store voucher and travel expenses.

Each focus group included between 4 and 9 parents, together with a facilitator and note taker. The focus group sessions followed the guidelines set out by Krueger and colleagues (1994)[17]. Participants were provided with a resume of the study's aims and objectives and given an opportunity to ask further questions prior to providing written informed consent. Participants were informed they could withdraw from the focus group at any time but, as their views would be audio-recorded along with other participants; their data could not be erased until transcription.

### Data collection

Participants were asked to complete a short, anonymous questionnaire detailing demographic details (by postcode) and ethnicity, information about family size, infant feeding history, parental weight and height. The facilitators offered parents help to complete the questionnaire, where appropriate. Each parent was provided with a set of ground rules prior to the focus group session. The facilitators were aided by a semi-structured topic guide (Table 2). The guide was revised following each focus group as issues important to the participants emerged. In addition to responding to the topics raised by the facilitators, participants were encouraged to express their own views and to lead the discussion in other directions within the remit of the research topic.

**Table 2:** Topic Guide for Focus Group Interviews

**Objective 1**. To explore perceptions of a good and/or appropriate size for an infant and to identify possible cultural influences
Can you describe your baby's size at birth and your thoughts about this?
How did your baby grow during the first few months of life? What are your thoughts about this?
Has anyone commented on the size of your baby? What did they say and how did you feel about this?
Has anyone's baby grown rapidly on the growth charts? If you are happy to do so can you describe this to us.
**Objective 2**. To explore parents' views about infants being identified as 'at risk' of childhood obesity by a health professional and to highlight potential barriers to implementation of early identification and prevention of obesity.
Has anyone's baby been identified as "at risk" of developing childhood obesity by a health professional? How did you respond at the time? What are your thoughts about it now?
If a baby was putting on too much weight at what point do you think a health professional should talk to the parents?
How do you think parents might feel if a health professional told them their baby was growing too fast?
Is it possible for a baby to be overweight?
How do you feel about preventing babies from becoming overweight?
Has anyone got any ideas about how to prevent childhood obesity? Where did these ideas come from?

**Objective 3**. To determine parents' approach to weaning their infants, including food choices, food restrictions, and beliefs about infant feeding practices and energy balance
Would anyone like to talk about how often they feed their baby and how much food their baby eats? Can you tell us how you decide when your baby has had sufficient to eat? What prompts you to offer your baby snacks?
When can you introduce solid foods to a baby?
Does anyone know what the Department of Health recommended age is for the introduction of solid foods?
Who do you listen to when it comes to taking advice about feeding your baby?
Can anyone recall being given advice from a health professional about a suitable diet for a baby under 1 year old?

The focus groups were audio recorded and transcribed verbatim. Transcription was performed immediately after each focus group to enable identification of individual parents. Participants were provided with an identification number on the transcribed interviews. Notes were used to track each participant's contribution.

### Data analysis

Data from the questionnaire was analysed using descriptive and frequency statistics facilitated by SPSS software[18]. The interview data were approached from an interpretative perspective. This involves the researchers looking beyond the actual spoken words in an attempt to understand the meaning behind the discussions[19]. All of the transcribed focus groups were entered into NVIVO 8.0[20]. Two researchers independently coded three of the focus group transcripts each using an inductive approach[19]. The codes were then arranged into related clusters to form a coding frame. The coding frame was then explored for linked and explanatory themes. A coding book was developed following the guidelines set out by Boyatzis (1998) which comprised codes, definitions and examples[21]. An inter-rater reliability check conducted on 10% of the coded data revealed 88% agreement. The coding frame was further revised between two coders and the main themes agreed. The data were then re-analysed as appropriate within the revised framework of themes and sub-themes.

## Results and Discussion

### Description of sample

Thirty-eight participants consented to take part in the study (n = 36 female, n = 2 male). Participants' ages ranged from 19-45 years (mean 30.1 years, SD 6.28). Body mass index (kg/m^2^) was calculated for each participant using self reported weight and height and (BMI < 25), with 12/38 classified as overweight (BMI 25-29.99) and 8/38 as obese (BMI >30) (Table 3).

**Table 3:** Description of parent participants

Gender	
Female	36 (94.7%)
Male	2 (5.3%)
**Age (years)**	
Mean (SD)	30.10 (6.28)
Range	19-49

**Ethnicity**	
White British	29 (76.3%)
White European	4 (10.5%)
Black - other	3 (7.9%)
White - other	1 (2.6%)
Asian - other	1 (2.6)

**Marital Status**	
Married	22 (57.9%)
Living with someone	10 (26.3%)
Single	6 (15.8%)

**Employment status**	
Employed full-time	17 (44.7%)
Employed part-time	12 (31.6%)
Unemployed	9 (23.7%)

**Occupation **[45]	
Managers and Senior Officials (1)	3 (7.9%)
Professionals (2)	5 (13.2%)
Associate Professional/Technical (3)	8 (21.1%)
Admin and Secretarial (4)	11 (28.9%)
Skilled Trades (5)	7 (18.4%)
Sales and customer services (7)	2 (5.3%)
Process, plant machinery operatives (8)	1 (2.6%)
Missing response	1 (2.6%)

**Body Mass Index (kg/m^2^)**	
Mean (SD)	26.61 (4.77)
Range	19.50-38.40
Number of parents who were overweight only	12 (31.6%)
Number of parents who were obese	8 (21.1%)

The mean age of the infants was 5.51 months (range 1-11 months) and most (27/38) were breast fed for at least a month (mean 4.48 months, SD 2.58, range 1-10 months) (Table 4).

**Table 4:** Description of family and infant characteristics

Number of children in family	
Mode	1
Range	1-5
**Age of infant (in months)**	
Mean	5.51
Range	1-11

**Gender of infant**	
Female	16 (42.1%)
Male	22 (57.9%)

**Breast fed (at all)**	
No	11(28.9%)
Yes	27 (71.1%)
**Length of time breast fed in months (n = 27)**	
Mean (SD)	4.48 (2.58)
Range	1-10

### Study themes

#### a) Parental concerns about breast milk, infant contentment and growth

##### Breast feeding and slower infant growth

First time mothers reported difficulties in establishing and maintaining breast feeding and felt that repeated weighing of their infants undermined their progress. This was particularly acute where an infant's weight was lower than anticipated on the growth chart trajectory.

It is extremely worrying though when you look at a baby and they look absolutely fine to you but then put them on the scales and they say that's wrong. Participant number 310

[Child's name] was 8lbs 3 and a half when she was born. I stopped breast feeding cos she wasn't putting on a bit of weight and then she just dropped and now she's following the line which she was on. Participant number 502

There was a strong belief amongst these parents that their breast milk supply was inadequate leading them to supplement their infant's diet with formula milk. This belief was apparent even in more affluent locality.

*I was breast feeding him but then I just could never produce enough milk so at fourteen weeks I had to give him a bottle because come six o'clock every night for two weeks on the trot I completely ran out of milk. Participant number 105*.

Parents suggested the decision to supplement breast milk with formula milk was often endorsed by health professionals. There was a belief amongst established breast feeders that this was an inappropriate intervention by health professionals and parents with this view described their vulnerability when trying to deal with this unwanted advice.

I wasn't going to give up but I was panicking every time they came expecting them to say you're going to have to put him on formula, until the weight gained. Participant number 310

In the UK, growth chart weight reference points have previously been based on norms for a mixture of breast and formula fed infants[22,23]. The problem of a mismatch between the reference points and breast fed infants' growth has been previously identified and new charts have now been introduced which set breast feeding as the norm[24]. Parents' accounts suggest that health professionals are not always aware of the difficulties monitoring the growth of breast fed infants using the old charts[22,23]. In the UK, the Royal College of Paediatrics and Child Health recently introduced training for health professionals on how to use the new charts[24] but at the time of data collection this may not have had an impact on parents' experiences.

I think it just adds panic and there's no point and they're not based on our figures in this country. Participant number 601

##### Perception that formula fed infants are more content

Parents who provide their infant with formula milk reported a sense of satisfaction based on having knowledge about the amount and timing of feeds. This enabled them to predict their infant's needs and made them feel less anxious about unexplained infant crying. This finding is supported by a larger study exploring the views of 503 British mothers' experiences of infant feeding. 405 respondents reported using formula milk and of these 76% were 'pleased to find a solution that made things easier', and 88% were, 'relieved that baby was being fed'[25].

Sleeps from eight to eight, and I can't complain, but I do think that the feeding we started to feed him and going from breast feeding, you never know what they get, and as soon as I put him on formula I felt more comfortable as a parent that okay, yeah you're having a good amount of milk I know how much you have had so I know you are not hungry when you are crying so when I started putting him to bed and he cried I thought well I know you are not hungry. Participant number 202

#### b) The belief that the main cause of infant distress is hunger is widespread and drives inappropriate feeding

##### Parents over-attribute infant distress as hunger

Parents preferred explanation for any changes in infant behaviour, such as unexpected or additional crying, or new night waking between 3 and 6 months, was that the baby was hungry. This resulted in the perception that infants were unsatisfied with a milk only diet and needed to be weaned onto solid foods. However, there are many other factors that could influence infant sleep patterns such as pre-conceptual stress[26], temperament[12] or minor illness. Baughcum and colleagues also reported that parents in their study believed infant crying was a sign of hunger[11]. A recent survey exploring maternal perception of infant feeding cues and pressuring feeding styles reported that 72% of mothers believed that infant crying is a sign of hunger[27].

I started to wean her because she started to wake up at night. Participant number 509

Parents reported weaning their infant earlier than the Department of Health's minimum age of six months,[28] which they considered a difficult goal to achieve. They reported being advised by a health professional to wean their older children at 3 or 4 months and having done so with no adverse consequences they saw no reason to treat their current infant differently.

I know there's more research into like the foods and the allergies and things like that but I also think it is hard when you have had a child already like you're saying yours at four months it's in your head isn't it that all the others were on food at four months. Participant number 402

These findings are supported within the literature. Baughcum and colleagues found that the low income mothers in their study weaned their infants prematurely; a pattern was identified which began with early introduction of rice and cereals and moved onto age inappropriate feeding of other solid foods[11].

##### Family and peers influence decision-making around infant feeding

Parents reported trying to refrain from age-inappropriate feeding but described family and peer pressure to top-up breast feeds with formula milk, to supplement formula milk feeds with rusks and to start weaning early.

My sister in law (name) her little baby (name) erm little girl she erm she's been having baby rice from four months and when she said to me 'you've only just started her on baby rice' and I said 'well yeah you know in the books now it doesn't say to start weaning until six months anyway', but she looked at me gone out, like 'oh no she's only been milk, she's only been fed by milk' Participant number 201

Parents believed that health professionals supported weaning earlier than the guidance suggested. They reported being advised by health visitors and nursery nurses that they could start weaning their infants onto baby rice and fruit at around four months but should wait until their infant was six months before feeding them more complex foods.

My health visitor said to wean between four and six months. Participant number 203

These results are not surprising since UK policy on recommended weaning ages has changed twice in the past ten years[28]. There seems to be little evidence to support the recommended weaning age of six months apart from a need to support exclusive breast feeding for six months as recommended by the World Health Organisation[29]. Parents are not convinced by this recommendation and UK health professionals require more evidence to support their advice around weaning ages especially in relation to formula fed infants.

Parents described at risk environments where their infant might be exposed unhealthy foods. A few parents mentioned private day nurseries and schools but the main concern stemmed from the negative influence of grandparents. Parents admitted it was grandparents' prerogative to indulge their grandchild but when this extended to family mealtimes they reported feeling they had lost control of their infant's diet.

It's like whenever the grandparents have got him it's like chocolate this and that and it's like when he's with me it's different it's fruit. Participant number 311

Baughcum and colleagues found low income parents were strongly influenced by their parents' ideas about infant feeding practices. Grandmothers encouraged the use of cereals in formula milk bottles and early weaning onto solid foods[11]. Our study findings suggest there is often conflict between parents and family members in relation to infant feeding. Baughcum and colleagues suggest that interventions to alter infant feeding practice may need to include the education of grandmothers,[11] and our findings support a family approach.

#### c) Rationalisation for infant's larger size

##### Parents of premature babies have a strong desire for rapid catch-up growth

Parents expressed a sense of fulfillment when their infants gained large amounts of weight according to the centile charts. In particular, parents of premature infants had a strong desire for them to catch up to perceived norms, which was endorsed by others in the group.

He was only 2lbs 2oz when he was born so he was tiny, tiny, there was nothing to him really. He's now seven months so he's caught up really quickly. Participant number 602

He's done really well then. Participant number 601

Yes he has, he's now seven months so he's caught up pretty, he's caught up really quickly. Participant number 602

Low birth weight infants are at risk of developing childhood obesity largely because of nutritional and other factors that arise antenatally[30]. However, there is some evidence that rapid catch up growth can be detrimental in terms of long term health outcome for low birth weight infants. Ibanez et al (2006)[31] report that small for gestational age children, with rapid weight gain between birth and two years, experience greater central adiposity and insulin resistance between ages 2-4 years, predisposing them to childhood overweight and obesity. Rapid weight gain during infancy has been associated with a higher prevalence of overweight and/obesity during the life course[3-5].

##### Parental preference for fatter infants

This theme was evident in groups conducted in both affluent and deprived localities but was only found in parents classified overweight or obese in this study. These parents were biased towards larger babies and believed that having a bigger infant was healthier; a finding also reported previously[11].

Running around big fatty things, they look gorgeous. Participant number 601

*I was quite happy that he was getting a little bit podgy. Participant 311*.

There was a view that articulating a preference for a bigger infant is less socially acceptable than it was in previous generations. Although a minority of parents suggested they preferred larger infants the general view was that this was a stronger preference amongst older family members.

My parents and my mother in law all think babies should be fat. Participant number 506

##### Shame and stigma in relation to overweight or obese infants

Some parents believed there was shame and stigma in having an overweight or obese infant. Parents with overweight or obese infants found it easy to justify their infant's size amongst themselves but feared criticism from others, such as health professionals, family or peers.

And sometimes my friends have like you know something's been said surreptitiously about her being a bit bigger on her tummy and I'm thinking I don't know what you're supposed to do. You do get hurt, you do take it personally. Participant number 601

#### d) Parental uncertainty about identifying and managing infants "at risk" of obesity

##### Reluctance to identify their infants as overweight

This theme was only present in the overweight/obese mothers. Their most favoured attribution for larger infants was family history and for those affected there was a sense of learned helplessness.

[Baby's name] was nine pounds two when he was born so he was a big boy and I was told that you know when I had my twenty week scan they were like oh erm yes he's quite a large baby and I mean my husband's like six foot four and I'm five foot nine. Participant number 103

There was a general acceptance that large parents produce larger babies. Mothers were quite defensive about any suggestion that their infant might be overweight or growing too quickly

There are other things they can look at to measure, the health, the good health of the child, not just the weight, as it is these days just because of the weight, weight, weight, it's really putting a strain on parents really. Participant number 305

Overweight and obese mothers did not articulate any concerns about the health risks associated with obesity, nor did they describe what they might do to prevent their infants from becoming obese children. It seems unlikely that the parents taking part in this study were unconcerned about obesity but they may have failed to acknowledge that their own infant was at risk. A survey conducted in the US with 83 parents of older children (4-8 years) found parents of overweight children had a similar level of concern about the health risks of obesity and similar knowledge of healthy eating to parents of normal weight children. However, parents of overweight children greatly underestimated their child's weight[32]. Baughcum and colleagues[13] also found that mothers of overweight infants were much more concerned about their infants being underweight than overweight. Etelson et al (2003) suggest that recognition and acknowledgement of childhood obesity is the first step towards tackling the problem[32].

##### Parental beliefs about overweight or obese infants

Parents were happy to talk about overweight or obese infants they knew in the community but were wary of what they said in the group, possibly through fear of causing offence to other parents present.

When I was visiting a nursery and I seen a overweight baby, baby couldn't even move things like that and it's a bit sad it was obviously overweight, so baby can be overweight. Participant number 204

I think it's a mums own opinion whether they think their own child's overweight. Participant number 307

There was a strong belief that an infant's weight levels out at one year and led to a view that health professionals should not be assessing infants as "at risk" and introducing management plans until after they have started physically moving around. This view was also expressed by UK health professionals in a study exploring their role in managing infants' at risk of developing overweight/obesity[33].

I think babies can be chunky but I think you never know which way it's going to go until they get to an age and then it's just looking at what do you feed your child. Participant number 202

##### Parental preference for continuity and sensitivity in health professional approach

Parents emphasised the way they would like to be approached by a health professional if their infant was gaining too much weight. Parents generally preferred to be approached by someone whom they knew and respected.

Quite nice for it to be someone that perhaps you've got a bit of a relationship with and I don't know I mean I saw a lot of the health visitor when [baby's name] was very little and would have welcomed her and did welcome any advice that she had for me and she I felt that she actually knew [baby's name] quite well. Participant number 104

There was a view that health professionals needed to be more explicit about infant overweight with parents.

If it was me I'd like someone to be quite blatant with me cos I think then I'd do something about it I think if someone is a bit half hearted about it and says oh well you know your baby could be overweight or could be obese I probably wouldn't do anything about it, but if someone said to me it'd probably make me step back and think you know what we need to stop doing that, I'll stop doing that so but then again I know it can be hurtful and you know upsetting but I think sometimes you need that to make a change in your baby's diet Participant number 201

Parents were not keen on the idea of restricting diet during infancy even for very large infants but they thought that that dietary content and quality could be explored by health professionals.

*It should be 'what are you feeding them' because then diet is probably a big thing, there's nothing you can do when they're under one, if they're hungry you have to feed them that's it, I can't see any other way round it. Participant number 311*.

There is no evidence to support these findings in relation to parents of infants who gain weight rapidly. However, it is well-established that health care recipients value continuity of care from a provider with whom they have an on-going relationship[34]. The value upon which parents place the relationship with their health provider needs to be considered when designing any intervention.

#### e. Intentions and behaviour in relation to a healthy lifestyle

##### Gaps in understanding about a healthy diet and the organic myth

Parents believed good eating habits started from a young age and were receptive to the idea of getting it right from the start.

I think it should happen before weaning personally, I think that because if you get baby with bad habits, you won't, it will be difficult to change it later. Participant number 101

Parents were able to articulate some of the features of a healthy weaning diet, however, there were gaps between what parents understood to be healthy and what is actually known to be healthy. Parents living in rural localities criticised the limitations of their local shops and complained about the distance to supermarkets which stocked pre-prepared organic baby foods which they perceived to be better than local fresh foods. Parents from all localities reported offering their infants snacks between meals. The explanations for doing so ranged from the experience of sharing food with the parent to using snacks as a way of keeping infants going until the next meal. Parents preferred the idea of a snack with its own packaging and hence reduced the need to take cutlery and/or find containers themselves when away from home. Emphasis was placed on the value of organic convenience food snacks.

She's having milk sort of that kind of fills in the gaps between mealtime but she also has erm the organic they're like crisps kind of finger foods that you get from the supermarket. Participant number 203

Parents wanted to do the best for their infants and some, including those on low incomes believed that to do so involved providing expensive, organic foods. However, a recent review commissioned by the Food Standards Agency found no additional nutritional benefit associated with organic foods when compared with conventional foods[35].

When you calculate what it costs to feed children [product name] food and the healthy food it is quite expensive and I think it's time consuming cooking for children. Participant number 202

You know if you put organic one hundred percent this and if it doesn't say one hundred percent should I not be giving it to her? And you've got to pay through the nose for it. Participant number 309

Younger parents, particularly those living in the deprived localities, reported being unable to produce home cooked foods either for themselves or for their infants.

My parents cooked, they cooked really nice home meals but I don't know how to do them. Participant number 203

They seemed anxious about trying to cook even the simplest weaning foods and fed their infants pre-processed weaning foods. These parents believed that such foods were better than anything they could cook.

##### Infants' need physical activity

One group of parents described the importance of encouraging early physical activity for infants even if that just meant taking them out of their buggies and giving them more scope to move.

There's a buggy thing, but they're sat in their buggies. Participant number 605

This group of parents felt that more could be done to encourage early physical activity.

There's not much advice, information around encouraging your baby's mobility. Participant number 604

There are anecdotal reports from health professionals that suggest that extensive use of buggies may increase the risk of childhood obesity[36]. There is also (unpublished) evidence that suggests the use of forward facing buggies may be stressful for infants[37]. Clearly more research is needed to explore early years' activity and obesity risk.

### Strength and limitations of study

The descriptive data, including parents' height and weight, was collected by parental self-report. Self-reported weight measures have been reported as inaccurate with trends for under-reporting for weight and BMI and over-reporting for height[38]. This may mean that the participants in our study were more overweight or obese than reported. Caution is needed, however, since all the female participants had given birth within the previous year and the weight reported may not reflect their typical weight. Qualitative methods were used to understand parent's explanations for their infant's size, growth and diet. The results represent an underlying social reality for the participants and generate various hypotheses which contribute to our understanding of the theory around infant feeding practices[39]. An attempt was made to include study sites with high and low rates of childhood obesity in order to recruit more parent participants whose infants may be at high risk of rapid weight gain. However, local and national data on rates of childhood obesity were incomplete at the time the study was set up and the IMD of the health centre was used as a proxy measure[16] to select four of the six study sites. The median IMD for each of the six study sites was retrospectively re-calculated using the parent participant's individual postcodes (Table 1). This revealed that in some of the study sites the parents who participated in the focus groups were living in more affluent locations than predicted. We cannot, therefore, say with certainty that the areas selected were those where infants were at greatest risk, or in the case of one area lowest risk. Participants' views were more homogenous than expected which may be due to a group effect. It may also have reflected that, despite the wide sampling, personal commonalities between the participants outweighed the impact of their respective geographic locations. Participants were volunteers and the details of those who were approached but did not participate were not collected. Some of the views expressed may, therefore, be different from non-participants in some meaningful way.

Both the researchers, who undertook the field work for the study, are qualified health visitors. The 'health-care-professional-as-researcher' is uniquely placed as they are already immersed in the field and have important insights into patient issues[40]. However, they can add a significant power imbalance and raise difficulties for participants in how free they feel to be open and/or critical[41]. At the time of the study the researcher were not practising and this information was shared with the participants. This research was undertaken in areas where the researchers have never practised and, therefore, could not have had a professional relationship with the parents.

## Conclusions

This study has shown how views of appropriate infant size and growth might influence parental feeding behaviours. Overweight and obese women in our sample preferred larger infants which has been previously reported[11]. Cultural and individual preferences for larger infants appear to influence some parents and they are implicitly adopting poor infant feeding practices. This finding has been previously established. A prospective observational study conducted on mother-infant dyads (n = 3768) in Denmark found that high maternal pre-pregnant BMI is associated with lower gestational weight gain, increased infant weight gain, less breastfeeding and earlier weaning[42]. When compared to normal weight women, overweight and obese women breast fed for shorter duration and weaned their infants earlier[42].

The study also revealed gaps in knowledge regarding good infant feeding practices, a finding which has been previously reported[43]. Parents also perceived gaps and inconsistencies in the information they received from health professionals. The knowledge of UK health visitors, general practitioners and practice nurses in relation to infant feeding has not been reported in the recent literature; therefore it is difficult to know for certain if this is the case. Advice from friends and family heavily influenced infant feeding choices particularly where there was parental uncertainty and/or anxiety. There were gaps in parental understanding of infant behavior, especially crying, and a commonly held view that distress was caused by hunger, which was endorsed by peers and grandparents, may lead parents to overfeed. Parents' over attribution of "hunger" as an explanation for infant distress is of concern. It appears that parents are not being informed, or not hearing advice about alternative explanations for infant cues. Education about this might need to look at a mother's emotional capacity to seek alternative explanations and well as general advice about the use of food to soothe infants.

This study revealed a number of barriers to early intervention with parents of infants at risk of developing obesity. Parents were uncertain about the management of overweight or obesity during infancy but were receptive to prevention prior to weaning. Clearly there is scope to improve infant feeding practices. Obesity prevention programmes need to provide better support for parents about appropriate infant feeding. In particular, this should focus on helping parents understand the physiology of breast feeding, how to differentiate between infant distress caused by "hunger" and other causes and the timing of weaning. Some parents also need guidance about how to recognize and prepare healthy foods and how to facilitate health physical activity for infants. However, interventions based on knowledge deficit models[44] can only partially tackle obesity prevention. Parents lives are complex and their feeding practices may have evolved as ways of coping with difficult lives and perhaps difficult infant temperament.

## Competing interests

The authors declare that they have no competing interests.

## Authors' contributions

SR, DN, NS, JS and CG participated in the design of the study and the funding application. SR was the Principal Investigator for this project, leading on the ethics application, project management, facilitation of focus groups and data coding and interpretation, PA was the researcher who recruited the participants, co-facilitated the focus groups, transcribed the interview data and helped with the data coding; DN and NS also helped with the data coding. CG formally matched the codes to the participants' quotes and helped with the data interpretation. SR, PA, DN, NS, JS, and CG have contributed to drafts of this manuscript and have read and approved the final copy.

## Pre-publication history

The pre-publication history for this paper can be accessed here:

http://www.biomedcentral.com/1471-2458/10/711/prepub
